# Reminiscent—1877 vs. 1896

**Published:** 1896-07

**Authors:** 


### REMINISCENT—1877 vs 1896.

### How are the Mighty Fallen!—Some Interesting History.

A propos of the recent action of the Texas State Medical Association in adopting the draft of a bill to regulate the practice of medicine, prepared by a special committee to be presented to the legislature as expressing the sentiments (?) of the medical profession of the State, which bill provides for a board of medical examiners, to be composed of six physicians, four homeopaths and two eclectics, it is recalled that this same Association—some of the same members now leading in the new departure, (1877) took strong ground against affiliation with homeopaths, and by resolution in effect denounced it as unethical, and beneath the dignity of a physician.

Judge Turner, of this, the Travis county district, appointed a district board of medical examiners, consisting of one homeopath, one eclectic and one regular physician. The regular physician was Dr. J. M. Ross, of Brenham—a distinguished physician and a prominent member of the State Medical Association. He accepted the appointment and served on the board. This action brought down upon the head of the honorable judge the maledictions of the medico; they were indignant beyond the power of words to express—and as regards the erring brother, who had so "lowered the dignity of his noble profession as to fraternize with quacks on a medical board," it found expression in a red hot resolution before the Travis County (this county) Medical Society, of which Ross was, I believe, a member. Whether or not Drs. McLaughlin and Wooten were the authors of the resolution is not recalled, and the record can not be found, but it is known that they took active part in the move, and were amongst those who brought the matter before the State Association at Galveston. The Travis County Medical Society denounced the action of Dr. Ross in serving on the board, as unethical. The State Medical Association met shortly afterwards at Galveston, and the matter of the resolution adopted by the Travis County Medical Society was brought up, and the State Association asked to endorse and sustain their action. There was a bitter fight made over it. Dr. Ross had the late venerable Ashbel Smith and Dr. Jno. H. Pope to defend him. Dr. Swearingen who, it seems, had been appointed with McLaughlin and Wooten, a committee from Travis County Medical Society, to present the matter, but who did not go down with the other delegates, was telegraphed for by them, and went down, and as will be seen, participated in the controversy.

Here is the record: We have before us the Transactions of the Texas State Medical Association, ninth annual session, 1877. Held in Galveston, April 3, 4, 5 and 6, and extract from the minutes made by the then Secretary Dr. W. A. East:

The President stated that a resolution, handed in yesterday, endorsing and approving the action of the Travis County Medical Society in refusing to treat with irregular practitioners, could be read:

*Resolved*, That the State Medical Association approve of the action of the Travis County Medical Society, in refusing to cooperate, in any professional manner whatever, with irregular practitioners in establishing a mixed Board of Medical Examiners to regulate the practice of medicine and surgery in the State.

Dr. J. B. Robertson addressed the Convention on the resolution. He had hoped that the members of the Convention would have given the subject serious study before taking final action upon it. That the adoption of the resolution would array the State Medical Association against the Constitution and laws of the State. That to pass the resolution would be to warn the district judges that they must either obey the laws they had sworn to enforce, or, ignoring them, fall back upon the Medical Society of the State for instruction and guidance in the discharge of their duties. That whenever and wherever attempts were made to suppress irregulars, they had been strengthened in their claims for support.

Dr. T. D. Wooten said he was surprised at the position taken by Dr. Robertson. His convictions and understanding of the issue raised induced him to believe that all the district judges, except one or two, were in sympathy with the stand taken by the Travis County Medical Society. His argument in vindication and support of the course taken by the Society, of which he was a member, was read. He said he had been an active participant in the matter under discussion from its beginning, and, in doing so, was prompted by no other motive than that having for its object the elevation of legitimate medicine.

Dr. J. H. Sears, of Waco. said he had hoped the resolution would pass without trouble, and thereby maintain the honor of the profession, and advance its cause. He regarded the organization as being based upon the high desire to elevate, rather than degrade, the standard of the profession. Thought it was beneath the dignity of the profession to treat with an error of any kind, and particularly an error that aimed its consequences against the safety and well being of the human race—that a defeat of the resolution would result in the disintegration of the Medical Association. Hoped that the resolution would pass—its defeat would invite popular disgust rather than excite popular confidence in the dignity and honor of the profession.

[Drs. Ashbel Smith and J. H. Pope defended Dr. Ross. Remarks omitted on account of length.]

Dr. Greenville Dowell read a resolution passed at the Philadelphia convention, touching the question under discussion.

Dr. Cupples said the question was one of vast importance to the profession, and condemned the sentiment that would defer it to a future time for adjudication. He complimented Dr. Ross for the manly stand he had taken; believed that he had acted conscientiously in all he had done. Would vote for the resolution. Said the light of truth and irresistible principles, that govern the regular practice, could never be eclipsed by the energies and efforts of all its enemies combined. Could see no excuse now for the Convention, in the shape the question had taken, to evade its adjudication, and concluded by saying that if the relationship with the American Medical Association was to be kept up, the Convention was bound to sustain the resolution.

Dr. Swearingen said that Dr. Pope's distinctions were too fine for his comprehension, but that Dr. Robertson's position was easily understood. The general was afraid that the Convention would precipitate a revolution upon the State. He had passed through one revolution and came out of it badly, and evidently dreaded a repetition of his past experience. That Dr. Ross, in his explanation of his position, had shown the patriot. He loved a patriot, but he regretted that Dr. Ross had forgotten that he was a physician when he assumed that character in the Travis county affair. He knew Dr. Ross well, and deep down in his heart he knew that none but the keenest instincts of gentlemanly feeling had a place. Had none but feelings of regret when he saw Dr. Ross on the examining board. He could not rejoice when such men as Dr. Ross, upon whose head the wings of time had already began to scatter its silver dust, and around whose brow honor had entwined its wreaths, went into the midst of the enemies of truth and aided them in carrying on a warfare with virtue.

Dr. Dowell said, in voting for the resolution, the Convention, opposes, not the law, but the unjust judge who had improperly executed it.

Dr. Smith moved that the resolution be postponed for six months. Motion sustained by a vote of 39 yeas, to 35 nays.

Adjourned until 2:30 o'clock, p. m.

[On the third day the subject was reconsidered, and] Dr. Denton said the vote, yesterday, postponing for six months, the consideration of the resolution endorsing the action of the Travis County Society was simply to set the time of its hearing at a period when there would be no one to hear it. He favored action that would do justice to that society, which simply asked to have the ethics of the profession sustained and vindicated. That what these gentlemen had done was in strict conformity with the rulings of the highest medical authority in the land. The talk of flying in the face of judiciary power and raising a revolution was devoid of argument. There were but two judges of the twenty-seven in the State, who had overridden the wants of the people, and placed irregulars on the examining boards; and said, to postpone action for six months would do the State Association more harm than to act on it promptly and at once.

Dr. Wooten said it was not the Travis County Society who were moving for a reconsideration of the vote. They had come before the Convention conscious of the rectitude of their cause, and had simply asked to be vindicated or condemned in what they had done, to resist all infractions of the rules of the profession.

Dr. Dowell said if the Convention endorsed Dr. Ross it could secure no recognition by the American Medical Association, and, further, if the resolution did not pass, there would be a new

State organization formed which would pass it. Asked the Convention for God's sake to pass the resolution.

Dr. Denton moved that the substitute of Dr. Robertson, and the amendment by Dr. Trueheart, be laid on the table.

Dr. Robertson called for the yeas and nays:

Dr. Smith said a straight vote would speedily reach the sense of the Convention.

Dr. Robertson withdrew his call.

Vote taken, and motion to table carried.

Dr. Truehart called for a division.

Motion again carried, on division.

Dr. Dowell moved that the original resolution (endorsing Travis County Society) be passed.

Carried amid applause.

---

THE WRITER, as is well known, has, for years, through the JOURNAL, and in every legitimate way, advocated organization of the medical profession of Texas, and a higher standard of education and of professional character. He has a deep and abiding interest in the advancement and welfare of the State Association, as well as a conviction that it will, despite numerous obstacles, yet become representative of the great body of rational practitioners, and that membership will, in time, be an honor to be coveted; that it will be what its founders intended —a grand body of scientific and ethical physicians. It is for this reason, as stated elsewhere, that we protest against the step it has taken in its zeal to secure medical legislation, as unwise and unethical. It is an error of judgment. We protest in the name and in the interest of the great body of physicians in this State, for it must be borne in mind that perhaps nine-tenths of them are not yet members of the State Medical Association. We do not believe they will endorse the action of that body in asking to be associated with irregulars on a medical board. I say "irregulars," for I honestly believe that there is not one amongst even the strongest advocates of this measure but who will admit that he considers homeopaths as irregulars,—as quacks; that "their sectarian title makes them quacks," and knowing that they only pretend homeopathy, and really essay the regular practice without preparation, pronounce them, as we do, "frauds." Yet, how inconsistent! they are willing to be placed in this relationship with them as equals,—as representatives of three "respective schools." It is stultification!

